# The Effect of Unethical Leadership on Employees’ Unethical Pro-Organizational Behaviour: A Meta-Analysis

**DOI:** 10.5964/ejop.16341

**Published:** 2026-05-29

**Authors:** Mohd Ferdaus Harun, Maisarah Mohd Taib

**Affiliations:** 1 Department of Psychology, International Islamic University Malaysia, Kuala Lumpur, Malaysia; Massey University, Palmerston North, New Zealand

**Keywords:** unethical leadership, unethical pro-organizational behaviour, meta-analysis

## Abstract

**Background:**

This meta-analysis examines the relationship between unethical leadership and employees’ engagement in unethical pro-organizational behaviours. When leaders lack moral integrity, it can undermine employees’ ethical decision-making. By synthesizing evidence across studies, this research aims to clarify the scale and impact of this link, informing interventions to mitigate the harmful effects of unethical leadership on employee behaviour.

**Method:**

A systematic literature search across five online databases identified 129 empirical studies published between 2014 and 2024. These studies investigated the relationship between unethical leadership and unethical pro-organizational behaviours, examining constructs such as exploitative leadership, authoritarian leadership, and abusive supervision. Only studies that were rated as having moderate or strong overall quality based on the QATQS risk of bias assessment were included. The meta-analysis employed a univariate random-effects model, with effect sizes calculated using Fisher’s Z coefficient.

**Results:**

A total of 20 effect sizes from 12 studies were included, involving 6,892 participants across five countries, four from Asia (i.e., China, Vietnam, Saudi Arabia, and Iran) and the United States. The overall effect size was positively significant, indicating that unethical leadership moderately increases employees’ unethical pro-organizational behaviours. The substantial heterogeneity suggests variability beyond chance. While the funnel plot and Egger’s regression test indicate potential publication bias in the included studies, Duval and Tweedie’s trim-and-fill method did not identify sufficient evidence of missing studies to warrant adjusting the effect size.

**Conclusion:**

Unethical leadership demonstrates a moderate positive effect on employees’ engagement in unethical pro-organizational behaviours. Leaders’ unethical conduct directly shapes this trend. However, potential mediating factors, such as a moral engagement, value-congruence, and moderating factors, such as organizational culture and ethical climate, may also influence these associations.

Interest in the impact of unethical leadership on employee behaviour has grown significantly in recent years, particularly regarding unethical pro-organizational behaviour (UPB). This form of behaviour, though intended to benefit the organization, violates ethical standards and can lead to serious long-term consequences. Despite the increasing volume of research on this topic, findings remain inconsistent. Some studies report strong positive associations between unethical leadership and UPB (e.g., Schuh et al., 2021; Shaw et al., 2020), while others suggest minimal or context-dependent effects (e.g., Luan et al., 2023). These inconsistencies underscore the need for a systematic synthesis of the literature to clarify the strength and nature of this relationship through meta-analytic methods.

Unethical leadership is theorized to influence employee behaviour by shaping organizational norms and offering moral justifications for unethical conduct (Hsu, 2022; Lian et al., 2022; Paterson & Huang, 2019). For example, when leaders express appreciation for ethically questionable actions, employees may develop a sense of psychological entitlement, increasing the likelihood of repeated unethical behaviour (Liu et al., 2020). These effects are often reinforced through trickle-down leadership processes, whereby behaviours exhibited by higher-level leaders cascade through organizational hierarchies to influence subordinate leaders and employees (Mayer et al., 2009; Misha & van Dijke, 2019). Empirical evidence suggests that various leadership styles (such as ethical, transformational, authoritarian, or abusive leadership) can shape the conduct of subordinate leaders, who subsequently influence employee attitudes and actions (Li & Sun, 2015; Mawritz et al., 2012).

Unethical leadership demonstrates a similar cascading pattern. When senior leaders engage in or condone unethical practices for the benefit of the organization, these actions may become legitimized and replicated by subordinates, thereby promoting UPB through mechanisms such as social learning (Fehr et al., 2019; Lian et al., 2022). For instance, morally questionable decisions by leaders can foster a climate in which UPB becomes normalized and imitated at lower organizational levels (Greenbaum et al., 2018). While the role of ethical leadership in guiding moral intuitions has been well-documented, research on how unethical leadership distorts moral judgement remains relatively underexplored (Gan et al., 2023).

Given these multifaceted influences, it is crucial to examine how unethical leadership contributes to organizational environments that rationalize or legitimize UPB. A focused meta-analysis can help reconcile conflicting findings in the literature and clarify the extent to which unethical leadership drives these behaviours across various organizational contexts. Although several meta-analyses have addressed unethical workplace behaviour more broadly, none has provided a targeted synthesis of the specific relationship between unethical leadership and UPB. For example, Kish-Gephart et al. (2010) investigated antecedents of unethical decision-making in general. However, their study is broader and conceptually distinct. Another related meta-analysis by Jiang and Xie (2024) focused on the outcomes of UPB, rather than its antecedents. Luan et al. (2023) conducted a meta-analysis examining 27 antecedents of UPB, including three related forms of unethical leadership (i.e., abusive supervision, authoritarian leadership, and leader UPB). However, their broad scope limited the number of studies available per predictor (*k* = 7), which in turn reduced the precision of their estimates.

In contrast, the present study offers a more focused and updated meta-analytic synthesis of the relationship between unethical leadership and UPB, drawing from a larger empirical base. As Riaz et al. (2016) argue, even when prior meta-analyses exist, additional analyses are warranted to address fragmentation in the literature and enhance the validity of cumulative findings particularly in light of renewed attention to ethical failures within organizational leadership.

## Employees’ Unethical Pro-Organizational Behaviours

Unethical pro-organizational behaviour (UPB) refers to actions undertaken by employees with the intention of benefiting their organization, despite violating ethical standards, societal norms, or legal regulations (Umphress & Bingham, 2011). This construct has garnered growing scholarly attention due to its potential for causing significant harm to both organizations and society at large (Zhang et al., 2018). Notably, leaders’ engagement in UPB can foster behavioural contagion among subordinates, particularly when employees identify strongly with their leaders and possess a low moral identity (Zhang et al., 2018). Furthermore, the presence of departmental egoistic norms and high organizational identification may enhance the perceived ethicality of UPB, thereby increasing employees’ likelihood of participating in such behaviour (Graham et al., 2020).

The consequences of UPB are multifaceted and often paradoxical. Although these behaviours may yield short-term gains for organizations, such as increased performance or loyalty, they frequently lead to long-term ethical dilemmas, reputational damage, and the cultivation of a toxic workplace culture (Liu et al., 2020). The normalization of unethical conduct in the name of organizational benefit gradually erodes trust and undermines the ethical foundation of organizations, creating a slippery slope toward increasingly severe forms of misconduct (Graham et al., 2020). Moreover, UPB can produce conflicting outcomes for employees, simultaneously facilitating positive work-to-family spillover while contributing to heightened work stress and interpersonal conflict (Chen et al., 2009).

Several theoretical frameworks have been employed to explain the antecedents and mechanisms underlying UPB. These include social exchange theory, moral disengagement, organizational identification, and cognitive dissonance. Social exchange theory suggests that employees may engage in UPB as a form of reciprocity, particularly when they perceive inequities or imbalances in their relationship with the organization (Umphress et al., 2010). In parallel, the theory of moral disengagement elucidates how individuals rationalize unethical conduct through cognitive strategies such as moral justification and euphemistic labelling, thereby preserving a positive self-concept despite transgressing ethical norms (Chen et al., 2021). These theories are critical in understanding how otherwise ethical individuals justify participation in UPB.

Organizational identification further amplifies the likelihood of UPB. Employees who strongly align themselves with their organization’s mission and values may justify unethical actions as necessary means to achieve organizational success (Cheng et al., 2022; Umphress et al., 2010). Additionally, situational stressors such as job insecurity and performance pressure exacerbate the risk of UPB, as employees may resort to unethical means to secure their positions or meet expectations (Elshaer & Azazz, 2021; Ghosh, 2023). This interplay between individual motivation and organizational context illustrates the complex dynamics that drive UPB.

Cognitive dissonance also plays a critical role in shaping employees’ experiences of UPB. Engaging in unethical acts to benefit the organization can generate internal conflict, leading to heightened role stress and a reduction in prosocial behaviours, such as helping colleagues (Guo & Wang, 2025; Zhang et al., 2018). Under high-performance pressure, this dissonance may further evolve into moral disengagement and even broader organizational deviance (Wang et al., 2025). The experience of such dissonance can have detrimental implications for employees’ well-being, contributing to both psychological strain and physical health issues (Shah & Lacaze, 2025).

Kish-Gephart et al. (2010) provide a comprehensive framework for understanding the antecedents of unethical behaviour by categorizing them into three domains: individual characteristics (“bad apples”), moral issue attributes (“bad cases”), and organizational contexts (“bad barrels”). At the individual level, factors such as low cognitive moral development, high Machiavellianism trait, an external locus of control, and low job satisfaction are associated with a higher propensity for unethical conduct. At the situational level, the perceived moral intensity of a dilemma shaped by the magnitude of harm, proximity to those affected, and the degree of social consensus plays a significant role in ethical decision-making. Organizationally, environments marked by egoistic ethical climates, weak ethical cultures, and the absence or poor enforcement of ethical codes of conduct are more conducive to unethical behaviour. These dimensions collectively underscore the complex and interrelated influences that drive UPB in the workplace.

Recent research has also explored various organizational and leadership factors contributing to UPB. Elements such as self-sacrificial leadership, strong leader identification, and prevailing organizational norms have been shown to shape employees’ willingness to engage in unethical acts for the organization’s benefit (Kish-Gephart et al., 2010; Yang et al., 2020). Understanding these mechanisms is essential for organizations seeking to mitigate UPB and foster ethical conduct. Developing targeted interventions requires a comprehensive appreciation of how individual, contextual, and organizational factors interact to influence this behaviour.

Despite growing interest in UPB, conducting research in this area presents significant challenges particularly within multinational organizational contexts. The complexity arises from the intersection of organizational culture and national cultural dimensions, both of which exert substantial influence on employee behaviour. For instance, organizations that emphasize stability over flexibility are more likely to foster UPB among highly committed employees (Fulmore et al., 2024). Moreover, national cultural dimensions such as high-power distance, collectivism, and masculinity have been identified as conducive to the prevalence of UPB (Mishra et al., 2025). An unethical organizational culture (UOC) further amplifies these risks, with ethical relativism mediating the relationship between UOC and employee engagement in UPB (Vem et al., 2023). These findings underscore the methodological and conceptual challenges in isolating variables across cultural and organizational contexts. They also highlight the imperative for multinational corporations to consider both organizational and national cultural frameworks when developing strategies to prevent UPB.

## The Influence of Unethical Leadership on Employees’ Unethical Pro-Organizational Behaviours

Unethical leadership, defined by behaviours that violate ethical norms and undermine employee well-being, has become increasingly prevalent in organizational contexts (Liu & Qiu, 2015; Treviño et al., 2014). It encompasses a range of destructive actions, including the exploitation of employees, the prioritization of self-interest over organizational objectives, and the abuse of power (Zhang & Xiao, 2020). Leaders engaging in such behaviours often advance personal agendas at the expense of their subordinates and the organization by embezzling resources, shirking responsibilities, or transferring illicit benefits (Zhang et al., 2023).

Unethical leadership significantly undermines employees’ psychological well-being by fostering emotional exhaustion, eroding trust, and diminishing their sense of empowerment (Brown & Mitchell, 2010; Zheng et al., 2021). Prolonged exposure to such behaviours generates stress and anxiety as employees struggle to reconcile personal moral standards with observed misconduct (Tepper, 2000). At the same time, it weakens psychological safety, discouraging employees from voicing concerns for fear of retaliation (Brown & Mitchell, 2010) and reducing their sense of agency (Benlahcene & Meddour, 2023; Zheng et al., 2021). Collectively, these effects cultivate a toxic climate that not only damages well-being but also paves the way for employees’ unethical pro-organizational behaviours (Treviño et al., 2014).

The influence of unethical leadership on employee behaviour is complex. Employees who perceive their leaders as unethical may adopt similar behaviours to align with perceived organizational norms or to secure their standing within the organization (Umphress & Bingham, 2011; Zhang et al., 2023). This may stem from a lack of ethical guidance, the belief that unethical conduct is necessary for success, or fear of reprisal for non-conformity (Hoyt et al., 2013). Additionally, employees may feel morally disengaged when they interpret unethical actions as necessary to meet leader expectations or organizational demands (Almeida et al., 2022).

Unethical leadership fosters a psychological environment in which UPB is facilitated through various cognitive mechanisms. A central process is moral disengagement, wherein individuals deactivate internal moral standards, justifying unethical actions without self-reproach (Bandura, 1999). Leaders model such rationalizations, enabling subordinates to legitimise behaviours such as deception or misinformation when framed as beneficial to the organization (Lian et al., 2022; Yan et al., 2024). Moral decoupling exacerbates this by allowing individuals to separate ethical evaluations from task performance, preserving their self-concept while engaging in misconduct (Fehr et al., 2019). In such contexts, UPB is reframed not as wrongdoing, but as loyalty or strategic necessity.

Social learning and role modelling also play a pivotal role. Based on Bandura’s (1977) social learning theory, employees emulate the behaviours of leaders, particularly when unethical conduct is rewarded or reinforced (Mayer et al., 2009; Nguyen et al., 2021). In hierarchical settings, displacement or diffusion of responsibility further dilutes personal accountability, especially when unethical leadership implicitly condones UPB (Liu et al., 2021; Rui & Qi, 2021). Collectively, these processes diminish ethical restraint and normalize UPB as a rational and defensible course of action.

These psychological processes align with various forms of unethical leadership identified in the literature. For example, exploitative leadership fosters moral disengagement and encourages UPB (Basaad et al., 2023), while authoritarian leadership displaces moral responsibility and cultivates a compliance-oriented climate (Liu et al., 2021; Rui & Qi, 2021). Abusive supervision, similarly, increases employees’ inclination to rationalize unethical behaviour (Yan et al., 2024). Leaders high in Machiavellian traits or those who model UPB (L-UPB) serve as role models, implicitly endorsing such conduct (Lian et al., 2022; Nguyen et al., 2021). When combined with cognitive mechanisms such as leader moral disengagement and moral decoupling, these leadership styles reinforce a toxic ethical climate and perpetuate the trickle-down effects of unethical conduct within organizations (Fehr et al., 2019; Song et al., 2021).

Moreover, perceptions of injustice and unethical organizational practices can intensify employees’ willingness to engage in UPB, either as a form of retaliation or a strategy to protect their status (Treviño et al., 2014; Zhang et al., 2023; Zhang & Xiao, 2020). Although leadership styles such as transformational or empowering leadership are often perceived as inherently positive, research shows they can inadvertently foster UPB by overemphasizing organizational goals and leader approval (Jiang & Xie, 2024). Practices that encourage autonomy and empowerment (Shaw et al., 2020) or emphasize high performance and competitiveness (Gilbert & Sutherland, 2013) may unintentionally promote unethical conduct. From a social cognitive theory perspective (Bandura, 1977) and through the lens of motivated moral reasoning (Tenbrunsel & Messick, 2004), such contexts heighten employees’ perceived responsibility for outcomes, and under pressure, employees may rationalize unethical acts as necessary for organizational success or protection. Consequently, unethical leadership influence is not always overt; it can be subtly embedded in well-intentioned practices that normalize or reward unethical actions when framed as organizational loyalty (Kalshoven et al., 2016), thereby triggering moral disengagement and legitimizing misconduct.

Extensive research establish that leadership characteristics significantly shape an organization’s ethical climate (Brown & Mitchell, 2010; Hassan et al., 2023). Deficiencies in ethical leadership foster a permissive environment for unethical practices. Benlahcene and Meddour (2023) argue that leaders lacking ethical competence often rely on manipulation, creating conditions conducive to unethical conduct among followers. Similarly, Zheng et al. (2021) found that unethical leadership diminishes employees’ psychological empowerment, thereby increasing their susceptibility to engage in UPB. These findings collectively highlight the central role of leadership in either mitigating or exacerbating unethical conduct within organizational life.

## Method

The present study was conducted in accordance with the Preferred Reporting Items for Systematic Reviews and Meta-Analyses (PRISMA Statements, Page et al., 2021).

### Eligibility Criteria

The eligibility criteria for this meta-analysis study include quantitative empirical studies that examine the relationships between unethical leadership concepts and employees’ UPB. Specifically, the analysis includes studies that report on concepts such as leader UPB, unethical leadership, authoritarian leadership, leader moral disengagement, abusive supervision, exploitative leadership, and leader other-oriented perfectionism, in relation to employees’ UPB. The review incorporates both dyadic data and self-report survey data. Studies are not restricted by location or geography and encompass all types of settings and contexts. Only published articles from 2014–2024 are considered eligible. Qualitative studies are explicitly excluded from this analysis.

### Information Sources and Selection Strategies

A systematic literature search was conducted across five online databases subscribed to by the researchers' institution: Scopus, Emerald Insight, JSTOR, ProQuest, and ScienceDirect. The search utilized Boolean techniques with specific search terms, including “*unethical leadership AND unethical pro-organizational behaviour*,” “*leadership AND unethical pro-organizational behaviour*,” “*supervision AND unethical pro-organizational behaviour*,” and “*leader AND unethical pro-organizational behaviour*.” These online databases provide a vast repository of peer-reviewed literature, ensuring the meta-analysis is based on high-quality and relevant studies while maintaining the transparency and reproducibility of the literature search strategy. The literature search was conducted between 22 October 2024 and 20 November 2024. The reliance on online databases aligns with other meta-analyses, such as those by Cuijpers et al. (2023), Doǧru (2022), and Rogers et al. (2023), which also used online databases for their studies.

The study employed two coders for the selection of relevant studies. All data were independently screened and reviewed by two coders. Agreement was achieved for 86.5% of the extracted data, with initial discrepancies resolved through discussions between the coders to determine study inclusion. For studies with persistent disputes, both coders expanded their screening process to include full-text analysis to ensure thorough evaluation and consensus.

### Data Extraction

For the meta-analysis, study details were systematically coded following a codebook (see Harun, 2026) developed based on the recommendations of Card (2012). Extracted data included information on sample country, sample setting (e.g., industry), sample size, measures for independent and dependent variables, means and standard deviations for both variables, effect sizes, and standard errors. The data were initially coded in Microsoft Excel file and later transferred to SPSS software Version 29 for meta-analysis. The primary coder independently extracted data after conducting a systematic literature search, ensuring thorough documentation and accuracy. A secondary coder reviewed the extracted data, achieving an agreement rate of 86.5% on the identified information. All initial discrepancies were addressed through detailed discussion and consensus between the coders, ensuring the reliability and validity of the data extraction process.

One meta-analytic study (Luan et al., 2023) was included because it offers a comprehensive quantitative synthesis of antecedents to employees’ UPB, providing effect size estimates unavailable in the primary literature. To prevent double-counting and circular reasoning, we applied a conservative coding protocol. The meta-analysis was treated as a single analytic unit, with only aggregated effect sizes extracted, and its primary studies were excluded from separate coding. We also cross-checked its primary study list against our database to ensure no dataset was duplicated. This approach maintained statistical independence while incorporating meta-analytic evidence that strengthened the robustness and generalizability of our findings.

### Study Risk of Bias Assessment

The methodological quality of the included studies was assessed using the Quality Assessment Tool for Quantitative Studies (QATQS, National Collaborating Centre for Methods and Tools, 2010), which evaluates six key domains: selection bias, study design, confounders, blinding, data collection methods, and withdrawals/dropouts. Each study was rated as strong, moderate, or weak in each domain, with an overall global rating determined based on these domains. QATQS also highlights the discrepancies between reviewers and final decision after reconciliation. Table B2, Appendix B summarizes the quality assessment results based on QATQS criteria.

Of the 15 studies identified during systematic literature search, 80% (*n* = 12) were rated as strong, 7% (*n* = 1) as moderate, and the remaining 13% (*n* = 2) rated as weak. Strong studies typically demonstrated robust sampling strategies, appropriate control for confounding variables, and reliable data collection methods and analysis. Studies rated as moderate generally employed non-representative sampling techniques and lacked detailed descriptions of their data analysis procedures. Weak studies commonly exhibited high risks of selection bias, inadequate adjustment for confounders, or insufficient reporting of statistical analysis. Consequently, the two studies rated as weak were omitted from the subsequent analysis.

### Sensitivity Analysis

To assess the impact of study quality on the meta-analytic results, a sensitivity analysis was conducted by excluding studies rated as moderate quality. The exclusion of this study did not significantly alter the pooled effect size for all studies, *Ź* = .282 (*Z* = 7.741, *p* < .001, 95% *CI* = .211 to .353), compared to after exclusion, *Ź* = .286 (*Z* = 7.524, *p* < .001, 95% *CI* = .211 to .360). This indicates that the results are not substantially influenced by studies of moderate quality. A sensitivity analysis was not feasible for weak-quality studies due to insufficient reported information (e.g., effect size, variance, standard error).

### Meta-Analytic Method

The present study conducted a univariate meta‐analysis using a random‐effects model, as it provides more conservative effect size estimates compared to fixed‐effects models. A random‐effects model was applied to account for both within‐study sampling error and between‐study heterogeneity, thereby enabling generalization beyond the included studies (Borenstein et al., 2010; Luan et al., 2023). Moderator or subgroup analyses were not conducted due to insufficient numbers of effect sizes per category, which can yield unstable estimates and increase the risk of spurious findings (Viechtbauer, 2010). Effect sizes were calculated using Fisher’s Z coefficient. For studies that did not report effect sizes, they were computed from the provided raw correlation (*r*), standard deviation (*SD*), and sample size. For studies that reported standardized beta weights, they were transformed into *r* scores using the formula proposed by Peterson and Brown (2005) and subsequently converted to Fisher’s Z following the guidelines by Borenstein et al. (2009). For studies that report multiple independent effect sizes (e.g., Fehr et al., 2019; Liu et al., 2021; Luan et al., 2023; Schuh et al., 2021; Yan et al., 2024), all effect sizes were included. To assess heterogeneity, the present study observed the *Q*-statistic and *I^2^*-statistic, which evaluate variability across effect sizes beyond what is expected by chance. Additionally, publication bias was examined through a combination of visual inspection of funnel plots, Egger’s regression test, and Duval and Tweedie’s trim-and-fill method to account for potential bias in the included studies.

The data underlying this meta-analysis are openly available in Open Science Framework at Harun (2026).

## Results

### Study Selection

**Figure 1:**
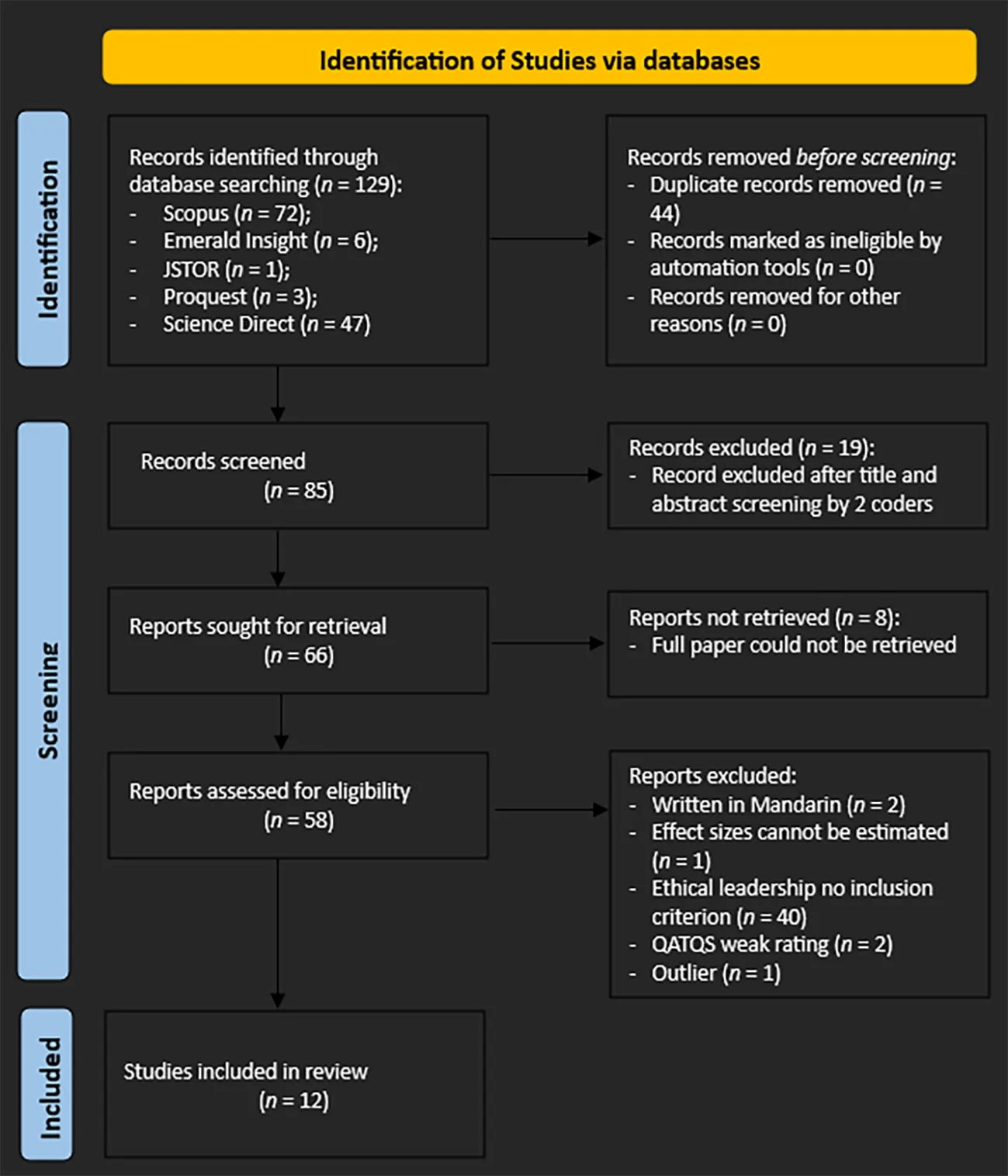
PRISMA Flow Diagram for Systematic Literature Search and Selection

A total of 129 studies were identified from five electronic databases subscribed to by the researchers’ institution (i.e., Scopus, Emerald Insight, JSTOR, Proquest, & Science Direct). After title and abstract screening, 66 studies were selected for full-text evaluation, and 13 studies met the eligibility criteria. However, one study was excluded due to an outlier, resulting in a final sample of 12 studies were included in the meta-analysis (see Figure 1 for meta-analytic flow diagram, Table 1 for individual study characteristics).

**Table 1:** Characteristics of the Included Studies

Study	Location	Industry	Research Design	Sampling	N	Mean Age	IV	DV
Basaad et al. (2023)	Saudi Arabia	Public sector Private sector	Cross-sectional survey	Convenience	208	20-29	Exploitative leadership	UPB
Fehr et al. (2019)	China	Manufacturing Sales Technology Financial services	Cross-sectional dyadic survey	Convenience	176	Supervisors: 36.92 Employees: 29.05	Leader UPB	UPB
	USA	-na-	Vignette-based experiment	Crowd-sourced	297	32.5	Leader UPB	UPB
	USA	-na-	Cross-sectional survey	Market research panels	281	55.6	Leader Moral Decoupling	UPB
Jiang et al. (2024)	China	Health care IT Retail Education Manufacturing	2 times-lagged design	Convenience	266	28.66	Leader Other-oriented Perfectionism	UPB
Lian et al. (2022)	China	Real estate	3 times-lagged design	Snowball	200	28.84	Leader UPB	UPB
Liu et al. (2021)	China	Software	Vignette-based experiment	Crowd-sourced	198	26-30	Authoritarian leadership	UPB
	China	Pharmaceutical	2 times-lagged design	Snowball	202	40	Authoritarian leadership	UPB
Luan et al. (2023)	-na-	-na-	Meta-analysis	-na-	1043	-na-	Abusive supervision	UPB
	-na-	-na-	Meta-analysis	-na-	377	-na-	Authoritarian leadership	UPB
	-na-	-na-	Meta-analysis	-na-	803	-na-	Leader UPB	UPB
Nguyen et al. (2021)	Vietnam	-na-	2 times-lagged dyadic design	Convenience + snowball	229	37.95	Leader UPB	UPB
Schuh et al. (2021)	China	Customer service	2 times-lagged dyadic design	Convenience	497	Leaders: 31-35 Employees: 26-30	Leader Moral Disengagement	UPB
	USA	-na-	Vignette-based experiment	Crowd-sourced	367	36.78	Leader Moral Disengagement	UPB
Shaw et al. (2020)	USA	IT	2 times-lagged design	-na-	175	27.75	Leader Moral Disengagement	UPB
Song et al. (2021)	China	-na-	Cross-sectional dyadic survey	Convenience + snowball	740	CEO: >40 Employees: >40	Leader Moral Disengagement	UPB
Wen et al. (2020)	China	Financial service	3 times-lagged dyadic design	Convenience	204	18-30	Leader UPB Leader Machiavellism	UPB
Yan et al. (2024)	China	-na-	2 times-lagged design	Crowd-sourced	629	<35	Abusive supervision Leader Moral Disengagement	UPB

*Note. UPB*: unethical pro-organizational behaviour; *na:* not reported; *N*: sample size; *IV*: independent variable; *DV*: dependent variable.

### Study Characteristics

The meta-analysis included 12 studies, with six studies reporting multiple effect sizes, resulting in a total of 20 effect sizes. All included studies examined the relationship between unethical leadership concepts and employees’ UPB. The analyses encompassed 6,892 participants across five countries, four from Asia (i.e., China, Vietnam, Saudi Arabia, and Iran) and the United States. The studies were conducted between 2019 and 2024. Most of the studies focused on leader UPB, authoritarian leadership, and leader moral disengagement, while other independent variables included abusive supervision, exploitative leadership, leader other-oriented perfectionism, and unethical leadership. The sample sizes ranged from 175 to 1,043 participants, with an average of 391. The studies primarily utilized time-lagged surveys, vignette-based experiments, and dyadic survey data matching leaders/supervisors with their subordinates.

A total of 20 effect sizes from 12 studies were included, involving 6,892 participants across five countries, four from Asia (i.e., China, Vietnam, Saudi Arabia, and Iran) and the United States

### Univariate Analysis

Effect sizes assessing the effects of unethical leadership on employees’ UPB ranged from *Ź* = -.79 to .54 across 20 samples. The overall mean effect size was significant and positive (*Ź* = .238, *p* < .001, 95% CI .116 to .360). Observation on the Forest plot identified one outlier study and was removed from the subsequent analysis. The overall effect size after the deletion has improved (*k* = 20, *Ź* = .282, *p* < .001, 95% CI .211 to .353). This overall effect size is equivalent to approximately *r* = .275. This is a modest in magnitude, carries meaningful implications for organizational practice. In applied settings, this result suggests that unethical leadership explains a notable proportion of variance in UPB which is enough to have tangible consequences when scaled across an entire workforce. Even a moderate association means that changes in leadership ethical conduct can meaningfully influence the prevalence of UPB carried out by the employees.

**Figure 2:**
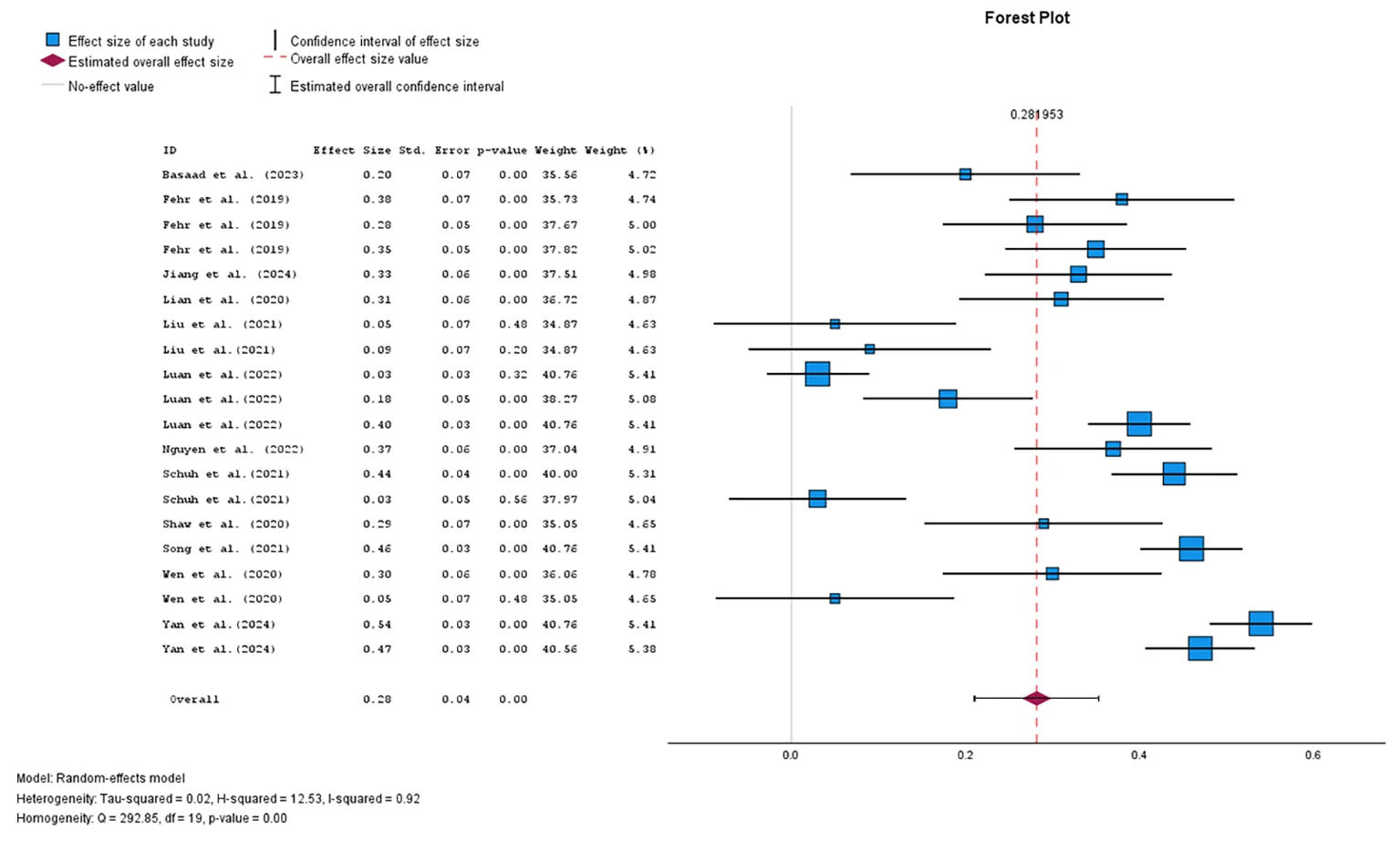
Forest Plot of Individual and Pooled Effect Sizes for the Relationship between Unethical Leadership and Unethical Pro-Organizational Behaviour

Heterogeneity analyses indicated substantial between-sample variability, suggesting that the observed variation in effect sizes reflects genuine differences between studies rather than random sampling error (*Q* = 292.85, *p* < .01, *I^2^* = 92; see Table 2 and Figure 2). This high heterogeneity implies that the strength of the relationships between unethical leadership and UPB is not uniform across contexts. Differences in leadership style operationalization, industry and cultural settings, sample composition, and measurement tools may all contribute to this variability. Consequently, the pooled effect size should be interpreted as an average across diverse conditions rather than a precise universal estimate. This highlights that practical applications should be sensitive to contextual factors rather than assuming a one-size-fits-all effect.

**Table 2:** Univariate Analyses of Mean Effect Sizes and Heterogeneity Tests

	Effect Size	95% CI	Heterogeneity
Outcome	*k*	*Fisher’s Z*	*Z*	*P*	Lower Limit	Upper Limit	*Q*	*df(Q)*	*P*	*I^2^*
Employees’ UPB	20	.282	7.741	< .001	.211	.353	292.85	19	< .001	92%

*Note. k* = number of studies, *Fisher’s Z* = transformed value of *r*, *Z* = z-score for test of statistical significance, *CI* = confidence interval, *Q* = Q-statistic of heterogeneity, *I^2^* = percentage of between-study variability.

### Publication Bias

**Figure C1:**
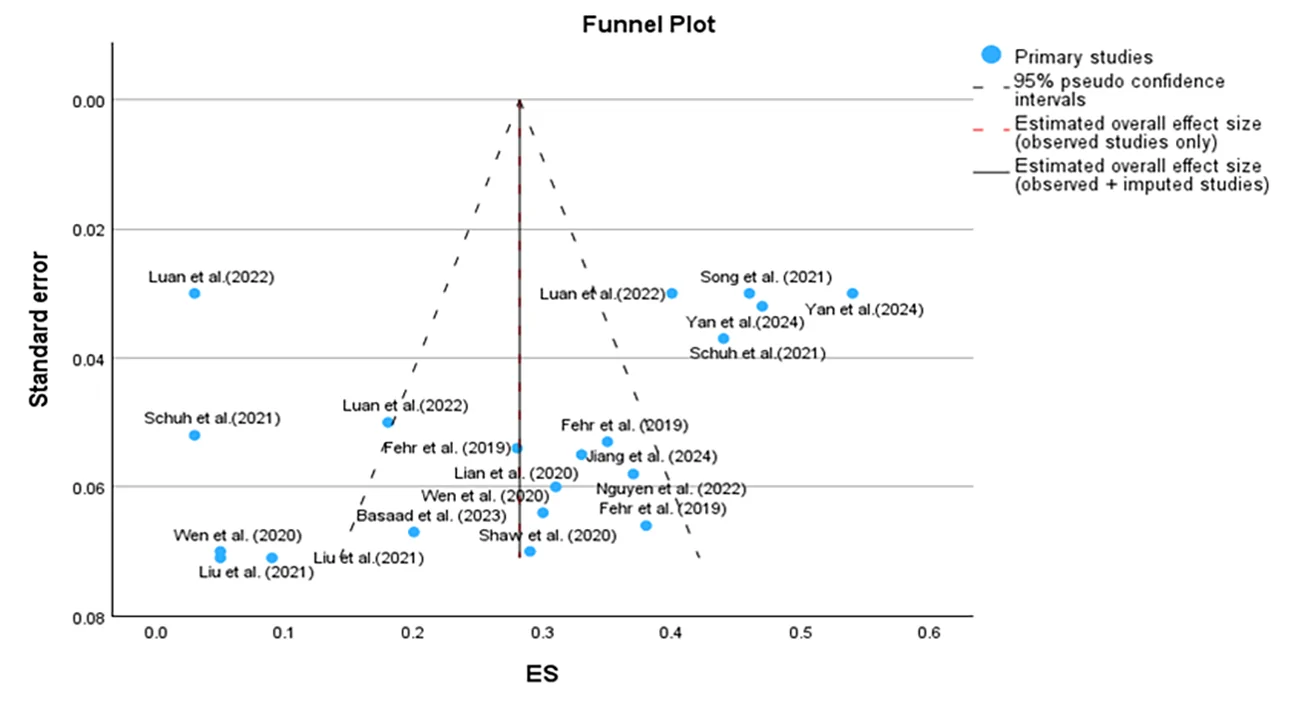
Funnel Plot Depicting Potential Publication Bias Based on Effect Size Symmetry

To assess the publication bias, a funnel plot was generated (Figure C1, Appendix C). Visual inspection revealed an asymmetrical distribution of effect sizes, suggesting that smaller studies exhibited greater variability and a tendency toward larger effect sizes. Egger’s regression test for funnel plot asymmetry was conducted to statistically evaluate this observation. The test revealed significant asymmetry, *t* = 4.676, *p* < .001, 95% CI .297 to .783, indicating a potential presence of publication bias, probably due to smaller studies with nonsignificant or smaller effect sizes being underrepresented.

To further explore this, Duval and Tweedie’s trim-and-fill method was applied. However, this analysis estimated zero potentially missing studies, and the adjusted overall effect size remained identical to the original analysis. This suggests that while Egger’s test detected asymmetry, the trim-and-fill method did not identify sufficient evidence of missing studies to adjust the effect size.

## Discussion

The present study conducted a univariate meta-analysis to examine the relationship between unethical leadership and employees’ unethical pro-organizational behaviour (UPB). Across 20 effect sizes from 12 empirical studies (*N* = 6,892; five countries), the aggregated mean Fisher’s *Z* was .282 (*r* = .275), indicating a small-to-moderate positive association that remained after sensitivity analysis. Substantial heterogeneity suggested variation in effect magnitude across contexts and study features. Although funnel plot inspection and Egger’s regression indicated possible publication bias, Duval and Tweedie’s trim-and-fill procedure confirmed the robustness of the estimate. These results suggest that unethical leadership fosters conditions in which UPB is normalised, potentially through mechanisms such as moral disengagement, social learning, and the trickle-down of unethical norms.

This suggests that unethical leadership has a statistically significant and meaningful, though not overwhelming, influence on employees’ UPB. While the relationship is robust and unlikely due to chance, the effect size highlights the complexity of human behaviour within organizational settings, where factors such as cultural norms, industry and organizational structures, sample composition, and measurement tools likely moderate or mediate this relationship (Fehr et al., 2019; Luan et al., 2023; Umphress & Bingham, 2011). Consequently, the pooled effect size should be viewed as an average across diverse conditions rather than a universal estimate (Luan et al., 2023; Mukherjee & Saritha, 2026). Even small to moderate effects can have substantial implications (Cohen, 1988), particularly in large organizations where the cumulative influence of leadership behaviours can profoundly shape workplace culture and employee conduct. Thus, unethical leadership is a significant driver of UPB, but its influence operates within a broader framework of organizational and contextual dynamics, underscoring the need for further investigation into these interacting factors.

This finding aligns with previous research, which has consistently highlighted the influential role of leadership behaviours in shaping organizational ethics and employee conduct (Antunez et al., 2024; Wang & Li, 2019). The analysis corroborates the theoretical assertions that unethical leadership practices, such as abusive supervision, authoritarian leadership, and moral disengagement, can foster an environment that encourages UPB among employees. These results emphasize the pervasive impact of leadership on employees’ behaviour, as suggested by theoretical frameworks like social learning theory (Moore et al., 2019), which posit that employees model the behaviours exhibited by their leaders.

Notable cases exemplify these dynamics. In the Volkswagen emissions scandal, employees manipulated test data to protect market position, fulfilling leaders’ organizational objectives but breaching ethical and legal norms, resulting in over USD 45 billion in penalties (Mukherjee & Saritha, 2026). In hospitality, unethical leadership prompted employees to conceal guest complaints, which further triggered UPB when moral awareness was low (Nosrati et al., 2024). Leaders high in organizational identification and moral disengagement were also found to condone employees’ UPB, rewarding staff for bending procurement rules (Schuh et al., 2021). Moreover, leader engagement in such behaviour produced contrasting outcomes. For example, under high Machiavellianism, subordinates replicated unethical practices, whereas under low Machiavellianism, they engaged in more organizational citizenship behaviours (Wen et al., 2020). Collectively, these cases illustrate how moral disengagement, social learning, and leader–follower value congruence drive the trickle-down and contagion effects of unethical leadership on employee UPB.

The small to moderate effect sizes in this meta-analysis indicate that contextual factors, such as cultural norms, industry dynamics, and organizational structures, may moderate the relationship between unethical leadership and employees’ UPB, reflecting genuine differences rather than random error (Aguinis et al., 2017).

Cultural norms strongly influence both perceptions and outcomes of leadership. In collectivist societies, authoritarian or directive styles may be interpreted as promoting group harmony, weakening their link to UPB, whereas in individualist cultures such behaviours may be perceived as illegitimate, amplifying the association (Dorfman et al., 1997; House et al., 2004; Pizam et al., 1997). Moreover, what counts as “unethical” varies across societies because legal and moral standards differ, shaping both leader behaviour and employee responses (Liu & Qiu, 2015; Vitell & Hidalgo, 2006). These findings align with research showing that leadership effectiveness depends on cultural congruence (Rockstuhl et al., 2011).

Industry conditions also play an important role. Highly competitive sectors such as finance, sales, and technology often tolerate or even reward rule-bending to meet performance targets, strengthening the unethical leadership–UPB link (Hassan et al., 2023; Zhang & Xiao, 2020). In contrast, highly regulated industries such as healthcare or aviation impose compliance systems and external oversight that constrain leaders’ ability to legitimize misconduct (Marmat et al., 2020). Organizational structures add another layer: rigid hierarchies can facilitate the top-down transmission of unethical norms, while flatter, decentralized designs provide more opportunities for ethical resistance (Kuenzi et al., 2020; Treviño et al., 2014).

Recognizing these potential moderators is critical for both research and practice. In collectivist, high power distance contexts, interventions should focus on empowering employees to speak up; in competitive industries, incentive systems must reward ethical conduct alongside productivity; and in regulated sectors, compliance can be reinforced through leadership accountability and cross-cultural ethics training. Integrating such contextual moderators, as emphasized in multidisciplinary frameworks (Koomson, 2022), can guide more effective, tailored interventions to disrupt the pathways linking unethical leadership to UPB.

### Strength and Limitations

A key strength of this study is its rigorous methodological design. The meta-analysis adhered to the PRISMA 2020 Statement (Page et al., 2021), using multiple high-quality databases, explicit inclusion criteria, and fully documented search strings (Table A1, Appendix A). Study quality was appraised via the QATQS, ensuring that only moderate- to high-quality empirical evidence informed the synthesis. A random-effects model with Fisher’s *Z* transformation was applied to accommodate substantial heterogeneity and permit generalisation beyond the sampled studies (Borenstein et al., 2009; Schmidt & Hunter, 2015). Methodological transparency was further demonstrated through publication bias assessment using funnel plots, Egger’s regression, and Duval and Tweedie’s trim-and-fill procedure (Sterne et al., 2011), enhancing the credibility and robustness of the findings.

Despite its methodological rigour, this meta-analysis has limitations. Substantial variation in effect sizes suggests that differences in sample characteristics, cultural contexts, and measurement tools may have influenced results. Although heterogeneity is common in meta-analyses, the univariate approach used here limits the ability to account for multiple sources of variability simultaneously (Riley et al., 2007), such as study design, cultural norms, and industry-specific factors. Cultural variability may further complicate comparability, as behaviours deemed unethical in one society may be viewed as normative in another, reflecting divergent value systems, leadership expectations, and ethical standards (Hofstede, 2001; House et al., 2004; Resick et al., 2006). Such differences can affect both the measurement and interpretation of unethical leadership and UPB, hindering direct cross-cultural comparisons (Dorfman et al., 2012; Tsui et al., 2007). Future meta-analyses should systematically code cultural background variables, such as Hofstede’s dimensions or GLOBE leadership scales, and test them as moderators to clarify the role of cultural context in shaping this relationship.

Secondly, the assessment of publication bias in this meta-analysis revealed potential concerns regarding the completeness and representativeness of the included studies. Both the funnel plot and Egger’s regression test indicated asymmetry, suggesting a possible underrepresentation of smaller studies with nonsignificant or weaker effects (Borenstein et al., 2009; Sterne et al., 2011). Although the trim-and-fill method estimated no missing studies, the observed asymmetry raises concerns that the overall effect size may be inflated. This pattern is consistent with the well-documented file drawer problem, wherein journals disproportionately publish studies with statistically significant results while null findings often remain unpublished (Rosenthal, 1979; Rothstein et al., 2005). As Rosenthal (1979) noted, such selective publication can distort the cumulative evidence base and lead to overestimated effect sizes. This bias is particularly relevant in the context of unethical leadership and UPB, where strong associations may be more appealing to publish, thereby shaping an incomplete or skewed understanding of the phenomenon (Franco et al., 2014).

The exclusion of unpublished studies further limits the comprehensiveness of this meta-analysis. Restricting the search to published literature may have overlooked valuable insights from grey literature such as dissertations, working papers, or internal organizational reports, which often contain nonsignificant results or employ diverse methodological approaches that could challenge or refine the conclusions (Hopewell et al., 2007; Song et al., 2010). Moreover, the search strategy relied primarily on electronic databases accessible through the authors’ institutional subscriptions and, to ensure methodological rigour, included only peer-reviewed journal articles. While this approach enhanced the quality and reliability of the evidence base, it inevitably excluded potentially relevant works indexed in other databases, such as PsycNET, and in grey literature repositories that may contain studies on unethical leadership and employees’ UPB. As Song et al. (2010) emphasised, the exclusion of unpublished and non-English studies can result in inflated estimates of effect sizes, thereby reducing the generalisability of findings.

### Recommendations for Future Studies

Future research should take proactive measures to address the above methodological limitations. Key strategies include systematically incorporating unpublished and grey literature to balance the evidence base, expanding database coverage to additional disciplinary repositories, and applying statistical corrections for publication bias, such as trim-and-fill procedures (Duval & Tweedie, 2000) or selection models (Vevea & Hedges, 1995). Greater transparency can also be achieved through pre-registration of study protocols (Nosek et al., 2018), open sharing of data and analysis scripts (Munafò et al., 2017), and journal policies that encourage the publication of null or mixed results. Replication studies conducted across diverse organizational and cultural contexts will be especially important for validating observed relationships and refining effect size estimates (Open Science Collaboration, 2015). Collectively, these practices can mitigate publication bias, broaden the evidence base, and strengthen the robustness of conclusions in the unethical leadership–UPB literature.

Beyond methodological improvements, future research should also deepen theoretical understanding by examining the moderating and mediating factors that shape the unethical leadership–UPB relationship. Unpacking mechanisms such as psychological safety, organizational norms, and individual personality traits can clarify the processes through which unethical leadership promotes UPB. At the same time, exploring moderators such as team dynamics, organizational size, and leadership tenure can highlight the contextual conditions that amplify or buffer these effects. Such analyses not only advance theory but also generate actionable insights for designing interventions that reduce the likelihood of UPB under unethical leadership.

Expanding the geographical and industrial scope of research is crucial to enhance the generalizability of findings. Cross-cultural studies are particularly valuable, as cultural norms and values shape perceptions of leadership and ethical behaviour (Zhu, 2007). Exploring underrepresented regions, such as ASEAN, Africa, South America, or the Middle East, can fill critical gaps and highlight cultural variations in the unethical leadership-UPB relationship. Similarly, future studies should investigate this dynamic across diverse industries, particularly those less represented in the literature, such as creative sectors, non-profit organizations, or small businesses. Understanding the influence of industry-specific norms and pressures on this relationship can help tailor ethical guidelines and interventions to different organizational contexts.

Additionally, employing a variety of research designs can address current methodological limitations and strengthen the robustness of findings. Longitudinal studies, for instance, can establish causal relationships by capturing how unethical leadership evolves and impacts employee behaviours over time. Experimental designs can offer controlled settings to test specific variables, while meta-analyses with broader leadership constructs can provide a more comprehensive understanding of the diverse ways in which leadership styles affect UPB. Investigating constructs such as servant leadership, transformational leadership, or emerging leadership styles can enrich the literature and uncover alternative pathways to mitigating unethical behaviours in organizations. By broadening theoretical and methodological approaches, future research can significantly advance the understanding of unethical leadership and its implications for workplace behaviour.

### Practical Implications

Organizations must take proactive measures to address the impact of unethical leadership on employee behaviours, beginning with comprehensive leadership development programs. These initiatives should focus on cultivating ethical leadership practices that actively discourage behaviours like abusive supervision and authoritarian leadership. Such training can mitigate the risks of unethical leadership influencing employees to engage in UPB, which often reflects a reciprocal response to leaders' unethical conduct. Moreover, ethics-focused educational programs should highlight the dangers of UPB, emphasizing that while these behaviours may appear beneficial to organizational goals in the short term, they can have long-term negative consequences for both individual employees and the organization as a whole. By fostering an understanding of the harm caused by unethical behaviours, these programs can reinforce the importance of ethical decision-making across all organizational levels.

To maintain ethical practices, organizations should broaden their ethics monitoring systems to encompass leaders, ensuring accountability extends beyond the employee level. Establishing comprehensive ethical guidelines that explicitly address leadership behaviours can cultivate a culture of transparency and integrity. Regulatory bodies and policymakers can further support these efforts by advocating for more robust ethical governance frameworks, particularly in industries where UPB are prevalent. Additionally, policies should incorporate strong whistleblower protections, empowering employees to report unethical practices without fear of retaliation. These safeguards can foster transparency and trust, ultimately creating an organizational environment that prioritizes ethical conduct and mitigates the risks associated with unethical leadership and UPB. Collectively, these measures can establish a holistic framework for ethical governance and sustainable organizational success.

## Supplementary Materials

**Table:** 

Type of supplementary material	Availability/Access
Data	
Leadership + UPB with no outlier 2024.11.23	Harun (2026)
Code	
Output 1	Harun (2026)
Output 2 - Sensitivity Analysis	Harun (2026)
Material	
No study materials provided.	—
Study/Analysis preregistration	
Study was not preregistered	—
Other	
Coding Book - Unethical Leadership + UPB	Harun (2026)

## Data Availability

The data underlying this meta-analysis are openly available in Open Science Framework at Harun (2026).

## Appendices

### Appendix A

#### Search Strings, Validation, and Inter-Coder Reliability

**Table A1:** Search Strings

Database	Search String	Filters Applied
Scopus	TITLE-ABS-KEY(("unethical leadership" OR "abusive supervision" OR "exploitative leadership" OR "authoritarian leadership" OR "leader moral disengagement" OR "leader other-oriented perfectionism" OR "leader unethical pro-organizational behaviour") AND ("unethical pro-organizational behaviour" OR "UPB")))	Article type = journal; Language = English; Year range: 2014–2024
Emerald Insight	("unethical leadership" OR "abusive supervision" OR "exploitative leadership" OR "authoritarian leadership" OR "leader moral disengagement" OR "leader other-oriented perfectionism" OR "leader unethical pro-organizational behaviour") AND ("unethical pro-organizational behaviour" OR "UPB")	Peer-reviewed; Language = English; Year range: 2014–2024
JSTOR	("unethical leadership" OR "abusive supervision" OR "exploitative leadership" OR "authoritarian leadership" OR "leader moral disengagement" OR "leader other-oriented perfectionism" OR "leader unethical pro-organizational behaviour") AND ("unethical pro-organizational behaviour" OR "UPB")	Peer-reviewed; Language = English; Year range: 2014–2024
ProQuest	TI,AB("unethical leadership" OR "abusive supervision" OR "exploitative leadership" OR "authoritarian leadership" OR "leader moral disengagement" OR "leader other-oriented perfectionism" OR "leader unethical pro-organizational behaviour") AND TI,AB("unethical pro-organizational behaviour" OR "UPB")	Peer-reviewed; Language = English; Year range: 2014–2024
ScienceDirect	TITLE-ABSTR-KEY(("unethical leadership" OR "abusive supervision" OR "exploitative leadership" OR "authoritarian leadership" OR "leader moral disengagement" OR "leader other-oriented perfectionism" OR "leader unethical pro-organizational behaviour") AND ("unethical pro-organizational behaviour" OR "UPB")))	Article type = journal; Language = English; Year range: 2014–2024

#### Validity of the Meta-Analysis

The validity of this meta-analysis was maintained through strict adherence to the Preferred Reporting Items for Systematic Reviews and Meta-Analyses (PRISMA) 2020 guidelines, prioritizing transparency, reproducibility, and methodological rigor. Key safeguards were implemented to address potential threats to validity at every stage from literature search to synthesis, ensuring that results are both trustworthy and replicable. Table A2 summarizes how validity procedures were addressed based on the selected PRISMA Statement Checklist (Page et al., 2021).

First, a comprehensive and reproducible search strategy was developed and documented, using multiple databases and Boolean search terms, with clear inclusion and exclusion criteria to minimize selection bias. Independent dual screening and consensus resolution reduced the risk of reviewer bias.

Second, the quality of included studies was systematically assessed using the Quality Assessment Tool for Quantitative Studies (QATQS), with weak studies excluded. A sensitivity analysis was performed to verify that findings remained stable when lower-quality studies were removed.

Third, statistical validity was ensured by standardizing effect sizes to Fisher’s Z, applying a random-effects model to account for both within- and between-study variance, and examining heterogeneity with the *Q* and *I^2^* statistics.

Finally, publication bias was assessed with funnel plots, Egger’s regression, and trim-and-fill analysis, while data transparency was upheld by making all extracted data available through the Open Science Framework (OSF). These measures collectively safeguard the internal, external, and statistical validity of the review, ensuring that its conclusions are well-founded and reproducible.

**Table A2:** Validity Procedures Based on PRISMA Checklist

Validity Criteria	PRISMA Item	Operationalization in this study	Page Number
Transparency of the search and selection process – ensures internal validity by making the process reproducible and unbiased.	Item 5 – Eligibility criteria	Clear inclusion/exclusion rules: quantitative empirical studies (2014–2024), specified unethical leadership constructs, exclude qualitative studies; prevents post hoc selection bias.	11
	Items 6 & 7 – Information sources & Search strategy.	Five databases searched (Scopus, Emerald Insight, JSTOR, ProQuest, ScienceDirect) using full Boolean search terms; search dates documented (22 Oct–20 Nov 2024); reduces risk of missing relevant studies.	11
	Item 8 – Selection process.	Two independent coders; 86.5% agreement; discrepancies resolved via discussion or full-text review; limits reviewer bias.	12
	Item 16 – Study selection.	PRISMA flow diagram provided showing numbers at each stage; supports reproducibility and transparency.	15
Quality and bias assessment of included studies – ensures only robust evidence is synthesized.	Item 11 – Study risk of bias assessment	QATQS applied; weak studies excluded from analysis; ratings reported in Table 1.	13 & Appendix B.
	Item 15 – Certainty assessment	Sensitivity analysis excluding weak-quality studies; findings remained stable, confirming robustness.	13
Robust synthesis and effect size handling – strengthens statistical validity.	Item 12 – Effect measures.	All effect sizes converted to Fisher’s Z; betas transformed to r, then Z; standardizes outcomes for comparability.	14
	Item 13 – Synthesis methods.	Random-effects model applied to account for within- and between-study variation; heterogeneity quantified with Q and I^2^.	14
Bias detection in meta-analysis results – identifies threats to validity from incomplete evidence.	Item 14 – Reporting bias assessment.	Funnel plot, Egger’s regression, and trim-and-fill method used to assess possible publication bias.	20 – 21 & Appendix C.
Data transparency – allows independent verification of findings.	Item 27 – Availability of data.	Full dataset available via OSF repository, enabling replication and secondary analysis.	14

#### Inter-Rater Reliability

Two independent coders screened titles, abstracts, and full texts against the inclusion criteria. Agreement was achieved for 86.5% of the data extracted, indicating strong reliability (Landis & Koch, 1977). Discrepancies were resolved through discussion and, where necessary, full-text re-evaluation.

### Appendix B

**Table B2:** QATQS Quality Assessment of the Selected Studies

	Assessment Criteria						
Study	*SB*	*C*	*B*	*DC*	*WD*	GR	D
Basaad et al. (2023)	Moderate	Strong	Strong	Moderate	-na-	Moderate	No
Fehr et al. (2019)	Strong	Strong	Strong	Strong	Strong	Strong	No
Gigol (2020)	Weak	Strong	Moderate	Weak	-na-	Weak	No
Gigol (2021)	Weak	Strong	Moderate	Weak	-na-	Weak	No
Jiang et al. (2024)	Moderate	Strong	Strong	Strong	Strong	Strong	No
Lian et al. (2022)	Strong	Strong	Strong	Strong	Strong	Strong	No
Liu et al. (2021)	Strong	Strong	Strong	Strong	Strong	Strong	No
Luan et al. (2023)	Strong	Strong	Strong	Strong	Strong	Strong	No
Nguyen et al. (2021)	Moderate	Strong	Strong	Strong	Strong	Strong	No
Nosrati et al. (2024)	Moderate	Strong	Strong	Strong	Strong	Strong	No
Schuh et al. (2021)	Moderate	Strong	Strong	Strong	Strong	Strong	No
Shaw et al. (2020)	Moderate	Strong	Strong	Strong	Strong	Strong	No
Song et al. (2021)	Moderate	Strong	Strong	Strong	Strong	Strong	No
Wen et al. (2020)	Moderate	Strong	Strong	Strong	Strong	Strong	No
Yan et al. (2024)	Moderate	Strong	Strong	Strong	Strong	Strong	No

*Note.* SB: selection bias; C: confounders; B: blinding; DC: data collection methods; WD: withdrawal and dropouts; GR: global rating; D: discrepancies between reviewers; na: not applicable.
